# Optimized Metabolomic Approach to Identify Uremic Solutes in Plasma of Stage 3–4 Chronic Kidney Disease Patients

**DOI:** 10.1371/journal.pone.0071199

**Published:** 2013-08-02

**Authors:** Henricus A. M. Mutsaers, Udo F. H. Engelke, Martijn J. G. Wilmer, Jack F. M. Wetzels, Ron A. Wevers, Lambertus P. van den Heuvel, Joost G. Hoenderop, Rosalinde Masereeuw

**Affiliations:** 1 Department of Pharmacology and Toxicology, Radboud University Nijmegen Medical Centre, Nijmegen Centre for Molecular Life Sciences, Nijmegen, The Netherlands; 2 Department of Physiology, Radboud University Nijmegen Medical Centre, Nijmegen Centre for Molecular Life Sciences, Nijmegen, The Netherlands; 3 Department of Laboratory Medicine, Laboratory of Genetic, Endocrine and Metabolic Diseases, Radboud University Nijmegen Medical Centre, Nijmegen, The Netherlands; 4 Department of Nephrology, Radboud University Nijmegen Medical Centre, Nijmegen, The Netherlands; 5 Department of Pediatrics, Radboud University Nijmegen Medical Centre, Nijmegen, The Netherlands; 6 Department of Pediatrics, Catholic University Leuven, Leuven, Belgium; University of Tokushima, Japan

## Abstract

**Background:**

Chronic kidney disease (CKD) is characterized by the progressive accumulation of various potential toxic solutes. Furthermore, uremic plasma is a complex mixture hampering accurate determination of uremic toxin levels and the identification of novel uremic solutes.

**Methods:**

In this study, we applied ^1^H-nuclear magnetic resonance (NMR) spectroscopy, following three distinct deproteinization strategies, to determine differences in the plasma metabolic status of stage 3–4 CKD patients and healthy controls. Moreover, the human renal proximal tubule cell line (ciPTEC) was used to study the influence of newly indentified uremic solutes on renal phenotype and functionality.

**Results:**

Protein removal via ultrafiltration and acetonitrile precipitation are complementary techniques and both are required to obtain a clear metabolome profile. This new approach, revealed that a total of 14 metabolites were elevated in uremic plasma. In addition to confirming the retention of several previously identified uremic toxins, including p-cresyl sulphate, two novel uremic retentions solutes were detected, namely dimethyl sulphone (DMSO_2_) and 2-hydroxyisobutyric acid (2-HIBA). Our results show that these metabolites accumulate in non-dialysis CKD patients from 9±7 µM (control) to 51±29 µM and from 7 (0–9) µM (control) to 32±15 µM, respectively. Furthermore, exposure of ciPTEC to clinically relevant concentrations of both solutes resulted in an increased protein expression of the mesenchymal marker vimentin with more than 10% (p<0.05). Moreover, the loss of epithelial characteristics significantly correlated with a loss of glucuronidation activity (Pearson r = −0.63; p<0.05). In addition, both solutes did not affect cell viability nor mitochondrial activity.

**Conclusions:**

This study demonstrates the importance of sample preparation techniques in the identification of uremic retention solutes using ^1^H-NMR spectroscopy, and provide insight into the negative impact of DMSO_2_ and 2-HIBA on ciPTEC, which could aid in understanding the progressive nature of renal disease.

## Introduction

The kidneys play an important role in maintaining total body homeostasis by facilitating the urinary secretion of both endogenous and exogenous waste products. Chronic kidney disease (CKD) affects approximately 10% of the adult population in developed countries. In half of these patients the diagnosis of CKD is based on the presence of a reduced kidney function (chronic renal failure; CRF). In CKD patients adequate renal clearance is compromised resulting in the accumulation of a plethora of uremic solutes [1]. Nowadays, over 140 uremic toxins have been reported, divided into three distinct classes based on their physico-chemical properties. It is well documented that uremic toxins accumulate in dialysis patients and several biomarkers of CKD have been identified [2]–[5]; yet, less is known about the retention of possible toxic solutes in other patients with a compromised kidney function. Herget-Rosenthal *et al*., reported that several uremic toxins are retained during acute kidney injury including β_2_-microglobulin, hippuric acid and 3-carboxyl-4-methyl-5-propyl-2-furanpropionic acid [6]. Furthermore, our group previously demonstrated that plasma levels of hippuric acid, indole-3-acetic acid, indoxyl sulphate and kynurenic acid are elevated in non-dialysis CRF patients compared with healthy controls [2].

Although the retention of uremic toxins in dialysis patients is widely studied, there is a large variation in blood levels reported for uremic toxins. These discrepancies can be due to differences in the study population with respect to diet, colonic microbial metabolism and endogenous metabolism [7], [8]. Moreover, precise determination of uremic toxin concentrations is cumbersome and dependent on the physico-chemical characteristics of these solutes, such as protein-binding, which can result in insufficient extraction of compounds from body fluids, leading to an underestimation of the true values [9], [10]. In addition, plasma is a complex mixture of proteins, molecules and ions that together can undergo a myriad of molecular interactions [11]. During ^1^H-nuclear magnetic resonance (NMR) spectroscopy, the abundance of proteins in complete plasma results in broad overlapping signals that obscure resonances of low-molecular-weight metabolites and quantification of these compounds is hampered due to T_2_-relaxation processes [11]. Therefore, deproteinization is required when studying small organic molecules in plasma, and it is required to optimize analytical techniques and/or sample preparation methods to obtain a reliable overview of uremic toxin levels in CKD patients.

In metabolomics studies, generally two analytical approaches are used: mass spectrometry-based methods and NMR [12]. Recently, Shah *et al.*, reported the plasma metabolite profiles of stage 2–4 CKD patients using gas and liquid chromatography coupled to mass spectrometry [13]. Therefore, we investigated whether NMR could be used as a complementary tool to elucidate novel biomarkers in kidney disease. The metabolic status of stage 3–4 CKD patients was determined using one-dimensional ^1^H-NMR spectroscopy following three previously described deproteinization strategies [11], namely ultrafiltration, protein precipitation via perchloric acid or via acetonitrile extraction.

## Materials and Methods

### Ethics Statement

The ethical committee of the Radboud University Nijmegen Medical Centre on research involving human subjects approved this study, and written informed consent was obtained from each patient and each healthy volunteer.

### Chemicals

All chemicals were obtained from Sigma (Zwijndrecht, the Netherlands) unless stated otherwise. Stock solutions of uremic toxins were prepared in milli-Q and stored at −20°C. The reference standard of p-cresyl sulphate, kindly provided by Prof. R. Vanholder (University Hospital Ghent, Belgium), was synthesized as a potassium salt as described previously [14].

### Patients and Sample Preparation

Blood samples were obtained from ten patients with CKD stage 3–4 (eGFR: 14–36 ml/min/1.73 m^2^) during regular check-up and four adult controls. Clinical characteristics of study subjects are listed in Table 1. None of the subjects had been fasting at the time of blood sampling. Blood was collected in an Heparin Vacutainer and was immediately centrifuged at 3,000 × g for 10 min. Subsequently, plasma was collected and stored at −20°C. Before analysis, each patient sample was deproteinized via three distinct methods: (1) ultrafiltration; plasma samples were deproteinized using a 10 kD filter (Sartorius). Before use, the filter was washed twice by centrifugation of water to remove glycerol. (2) perchloric acid (PCA) extraction; 100 µl of 20% (v/v) PCA was added to 500 µl plasma, samples were then vortexed and placed on ice for 5 min. Next, samples were centrifuged at 12,000 × g for 3 min and the clear supernatant was used for spectroscopy. (3) acetonitrile extraction; 1.5 ml of acetonitrile was added to 0.5 ml plasma, mixed thoroughly, followed by centrifugation (3,000 × g for 5 min). Subsequently, the supernatant was dried by heating at 40°C under N_2_ flow and finally resuspended in 700 µl milli-Q. The control samples were deproteinized via ultrafiltration or acetonitrile extraction. Following protein removal via the different methods, 20 µl of 20.2 mM trimethylsilyl-2,2,3,3-tetradeuteropropionic acid (TSP, sodium salt) in ^2^H_2_O was added to the sample, providing a chemical shift reference (δ = 0.00), a concentration reference and a deuterium lock signal. The pH of the ultrafiltrate was adjusted to 2.50±0.05 with concentrated hydrogen chloride. Finally, 650 µl of the sample was placed in a 5 mm NMR tube (Wilmad Royal Imperial).

**Table 1:** Characteristics of study subjects.

	Patients	Controls^a^
Number	10	4
Age (years)	55±12	40±12
Women (%)	33	50
Urea (mmol/l)	20±9	ND
Creatinine (µmol/l)	227±56	20–90
Albumin (g/l)	37±4	ND
eGFR (ml/min/1.73 m^2^)^b^	26±7	ND

Values are shown as mean ± SD. ND, not determined.

a Control metabolite levels were similar as compared to an established database (n = 50) from the Radboud University Nijmegen.

b eGFR was calculated using the Modification of Diet in Renal Disease (MDRD) equation (www.nkdep.nih.gov).

### One-dimensional ^1^H-NMR Spectroscopy

Plasma was measured at 500 MHz on a Bruker DRX 500 spectrometer equipped with a triple-resonance inverse (TXI) ^1^H [^15^N, ^13^C] probe head and equipped with x,y,z gradient coils. ^1^H spectra were acquired as 256 transients in 32K data points with a spectral width of 6002 Hz. Sample temperature was 298 K and the H_2_O resonance was pre-saturated by single-frequency irradiation during a relaxation delay of 10 s, and a 90° excitation was used. Automated tuning and matching (ATMA) and shimming (Topshim) was performed on all plasma samples. The resonances from the metabolites in Table 2 and the TSP singlet (nine equivalent protons) were fitted semi-automatically with Lorentzian line shapes. The concentration of the metabolites was calculated from the relative integrals of the fitted lineshapes using the known concentration of TSP.

**Table 2:** ^1^H resonance assignments and plasma concentrations of uremic solutes in stage 3–4 CKD patients.

Metabolite	Peak no.^a^	C_u_ (µM)	C_max_ b (µM)	Control (µM)	Literature^c^ (µM)
1-Methylhistidine	3	34±26	87	<5	4±8
3-Methylhistidine	4	38±25	89	ND	2.7 (0–6)
Hippuric acid		134±111	357	ND	3 (0–5)
p-Cresyl sulphate		289±132	552	ND	15±9
Creatinine	1,2	590±276	1143	20–90	72 (57–93)
Dimethyl sulphone	7	51±29	108	<30	9±7
2-Hydroxyisobutyric acid	9	32±15	61	ND	7 (0–9)
*N,N*-Dimethylglycine	8	23±11	46	<5	2.6 (1.8–3.7)
trigonelline		28±24	76	ND	ND
Pseudouridine		48±15	79	ND	3.2±1
Betaine		83±34	150	<50	34.6 (24–42)
*myo*-Inositol	5	499±170	838	ND	30 (21–49)
Dimethylamine		17^d^	NA	ND	3.3±1.5
Trimethylamine *N*-oxide	6	88±42	172	ND	38±20

Values are shown as mean (C_u_) ± SD or range (µM) and maximal uremic concentration (C_max_,). ND, not detected; NA, not applicable.

a Numbers correspond to peaks in Fig. 3.

b Hypothetical C_max_ calculated as C_max_ = C_u_ +2 SD, as previously described [2], [3].

c Data obtained from the Human Metabolome Database (www.hmdb.ca) [42].

d Only detected in one patient.

### Cell Culture

The human conditionally immortalized proximal tubule epithelial cell (ciPTEC) line was generated as previously described by Wilmer *et al*. [15]. The cells were cultured in phenol red free DMEM/F12 medium (Gibco/Invitrogen, Breda, the Netherlands) containing 10% (v/v) fetal calf serum (MP Biomedicals, Uden, the Netherlands), insulin (5 µg/ml), transferrin (5 µg/ml), selenium (5 ng/ml), hydrocortisone (36 ng/ml), epithelial growth factor (10 ng/ml), and tri-iodothyronine (40 pg/ml) at 33°C in a 5% (v/v) CO_2_ atmosphere. Propagation of cells was maintained by subculturing the cells at a dilution of 1∶3 to 1∶6 at 33°C. For experiments, cells were cultured at 33°C to 40% confluency, followed by maturation for 7 days at 37°C. Experiments were performed on the cells between passages 30 and 40, during which proximal tubule characteristics, such as albumin uptake and phosphate reabsorption, were maintained [15].

### Flow Cytometry

In this study, flow cytometry was used to study both cell viability and the expression of vimentin, a mesenchymal cell marker. ciPTEC were seeded at 40% confluence in 12-well plates and allowed to adhere over night at 33°C followed by maturation for 7 days at 37°C, before being treated for 48 h with clinically relevant uremic toxin concentrations. In addition, ciPTEC were also exposed for 48 h to 1 mM 1-methylhistidine, 3-methylhistidine (both as negative control) or indoxyl sulphate as a positive control. After incubation, cells were harvested using trypsin-EDTA and centrifuged at 600 × g during 5 min. Subsequently, supernatant was removed and the cell pellet was resuspended in 100 µL PBS containing 1 µL mouse-α-human Vimentin-PE (Abcam, Cambridge, UK) followed by 30 min incubation at RT. Samples were acquired with a BD FACSCalibur (Becton Dickinson, Breda, the Netherlands) using channel FL-2. Analysis was performed using Flow Jo software (TreeStar, Ashland, USA), gating on live cells.

### High-performance Liquid Chromatography (HPLC)

HPLC was used to measure UDP-glucuronosyltransferase (UGT) activity via the glucuronidation of 7-hydroxycoumarin (7-OCH), as described previously [16], [17]. Following exposure to uremic toxins at clinically relevant concentrations for 48 h, ciPTEC were incubated with 10 µM 7-OCH for 3 h at 37°C. Before chromatography, an aliquot of culture medium was collected and centrifuged at 12,000 × g for 3 min and 50 µl of the supernatant was injected into the HPLC-system (Spectra-Physics Analytical, Spectrasystem SCM400). To measure 7-OCH and 7-OCH glucuronide (7-OCHG) the HPLC was equipped with a C18 HPLC column (GraceSmart RP 18 5u 150 x 4.6 mm; Grace, Breda, the Netherlands). Separation was performed at a flow rate of 1 ml/min with eluent A (95% (v/v) H_2_O, 5% (v/v) methanol and 0.2% (v/v) acetic acid) and eluent B (50% (v/v) H_2_O, 49% (v/v) acetonitrile and 1% (v/v) tetrahydrofuran) under the following gradient conditions: 0–3 min, 80–50% eluent A; 3–8 min, 50% eluent A; 8–9 min, 50–80% eluent A; 9–14 min, 80% eluent A. The compounds were detected at a wavelength of 316/382 nm. Standards of the compounds were also run in order to quantify the amount of metabolites found in the samples. Acquired data were processed with PC1000 software (Spectrasystem).

### 3-[4,5-dimethylthiazol-2-yl]-2,5-diphenyl tetrazolium bromide (MTT) Assay

Mitochondrial succinate dehydrogenase activity was assessed using the MTT assay. ciPTEC were cultured in a 96 well culture plate and exposed to DMSO_2_ or 2-HIBA for 48 h. Next, medium was removed and 40 µl preheated (37°C) MTT-solution (5 mg 3-[4,5-dimethylthiazol-2-yl]-2,5-diphenyl tetrazolium bromide/ml ciPTEC medium) was added and incubated for 4 h at 37°C. Afterwards, MTT-solution was removed, followed by the addition of 150 µl DMSO to dissolve produced formazan crystals. The extinction of the solution was measured at 570 nm using a Benchmark Plus Microplate Spectrophotometer (Bio-rad).

### Statistics

Statistics were performed using GraphPad Prism 5.02 via one-way analysis of variance (ANOVA) followed by Dunnett's Multiple Comparison Test. Differences between groups were considered to be statistically significant when p<0.05. The software was also used to perform linear regression analysis and correlation analysis (Spearman and Pearson). Raw data files are available upon request.

## Results

### Influence of Deproteinization on ^1^H-NMR Spectra

**Figure 1:**
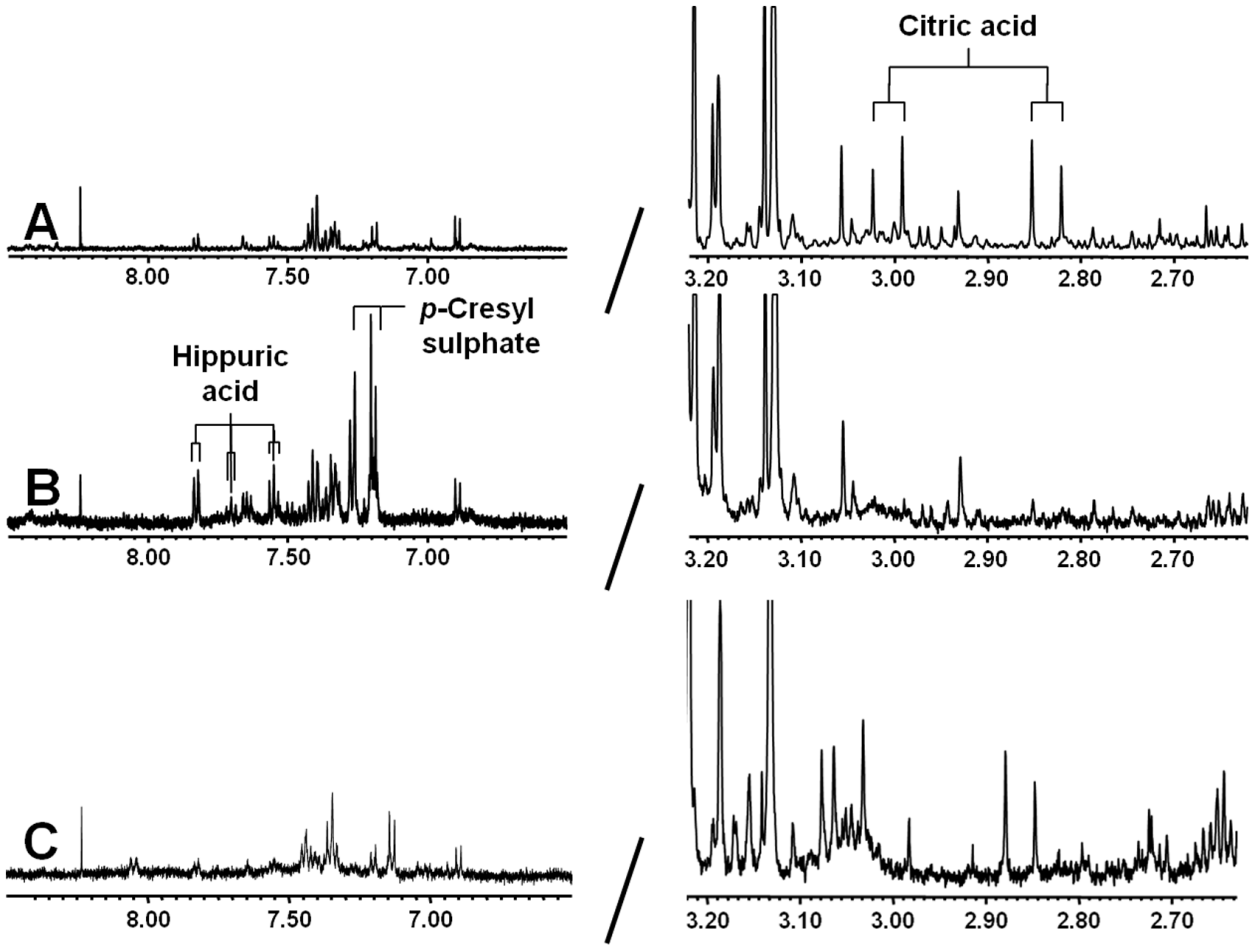
Comparison of deproteinization methods. 500 MHz ^1^H-NMR spectrum of plasma from CKD patient following (**A**) ultrafiltration, (**B**) acetonitrile precipitation or (**C**) PCA extraction.

**Figure 2:**
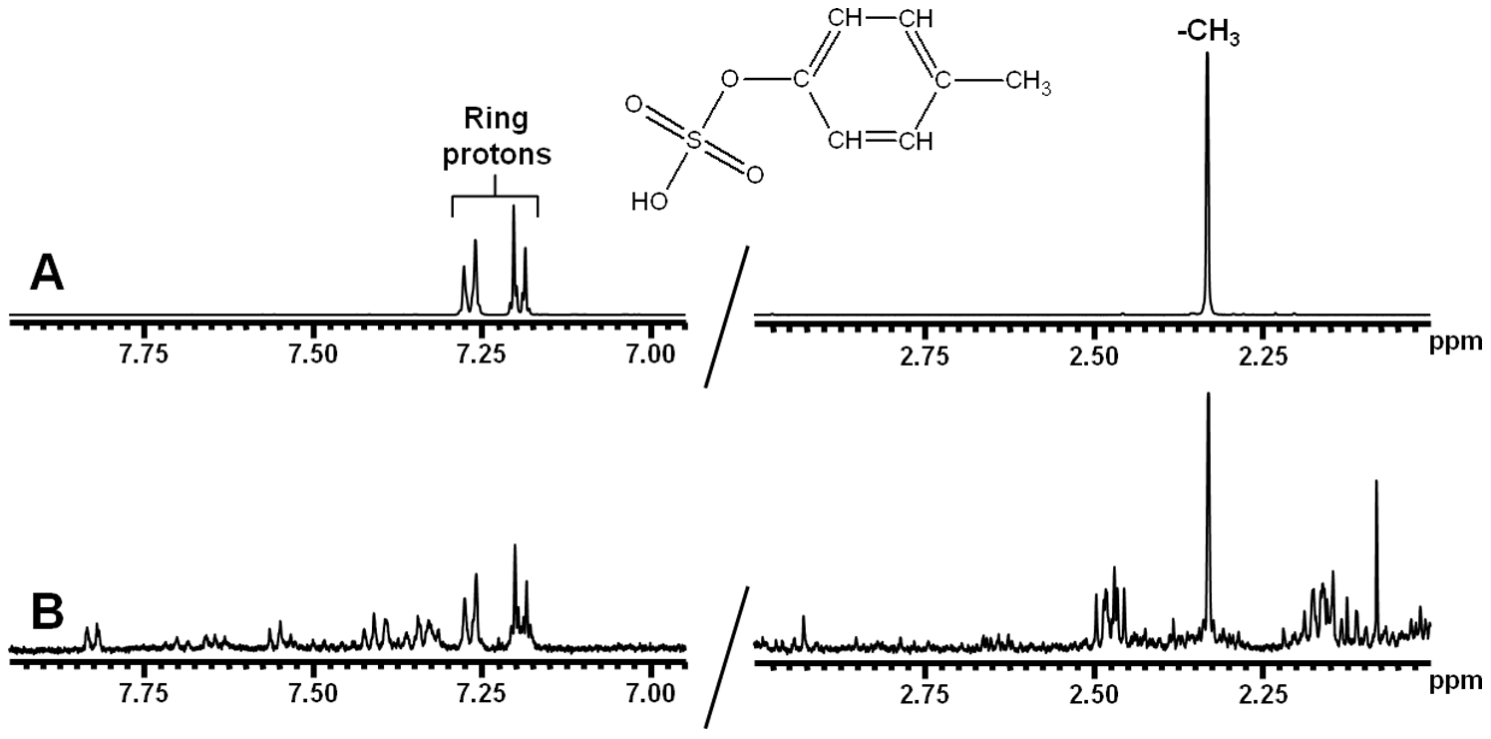
500 MHz ^1^H-NMR spectra: region δ 7.00–7.80 and 3.00–2.00. (**A**) Authentic reference solution of p-cresyl sulphate with chemical structure and assignments. (**B**) Plasma (acetonitrile precipitation) from CKD patient. The spectrum shows resonances of p-cresyl sulphate. These resonances were not observed in plasma from controls.

Fig. 1 shows the deproteinized ^1^H-NMR plasma spectrum following ultrafiltration (Figure 1A), acetonitrile precipitation (Figure 1B) and PCA extraction (Figure 1C). Citric acid is clearly detected following ultrafiltration with a symmetrical quadruplet, generally referred to as an AB-system, at 2.94 ppm; whereas, resonance signals are low in the spectral region δ 7.00–8.00 (Figure 1A). In contrast, high-resonance signals were observed in this part of the ^1^H-NMR spectrum after acetonitrile treatment (Figure 1B). Hippuric acid showed a triplet at both 7.54 ppm and 7.62 ppm, and a doublet at 7.82 ppm. The other resonance signals were assigned to p-cresyl sulphate. Moreover, Figure 1B also shows that the citric acid peak observed following ultrafiltration is lost by acetonitrile deproteinization. Furthermore, PCA extraction resulted in an overall decreased sensitivity, a poor signal-to-noise ratio and shifts in peak position (Figure 1C), making this method unsuitable for metabolite identification and quantification. To our knowledge, this is the first report to demonstrate the presence of p-cresyl sulphate in plasma using ^1^H-NMR spectroscopy. Therefore, we aimed to verify the identity of the detected metabolite using a reference standard. ^1^H-NMR of the authentic compound p-cresyl sulphate in H_2_O at pH 2.5 (Figure 2A) showed a singlet at 2.33 ppm (CH_3_ group) and a doublet at both 7.19 ppm and 7.27 ppm (aromatic ring protons). A similar resonance profile was observed in acetonitrile-treated plasma from a CKD patient (Figure 2B), indicating that p-cresyl sulphate is indeed retained in patients with kidney failure. The absence of both hippuric acid and p-cresyl sulphate in plasma following ultrafiltration is most likely due to the strong protein binding of these solutes. Thus, ultrafiltration and acetonitrile extraction are complementary deproteinization strategies and both methods are required to obtain a clear overview of the metabolic status of non-dialysis CKD patients.

### Accumulation of Uremic Toxins in Patients with CKD Stage 3–4

**Figure 3:**
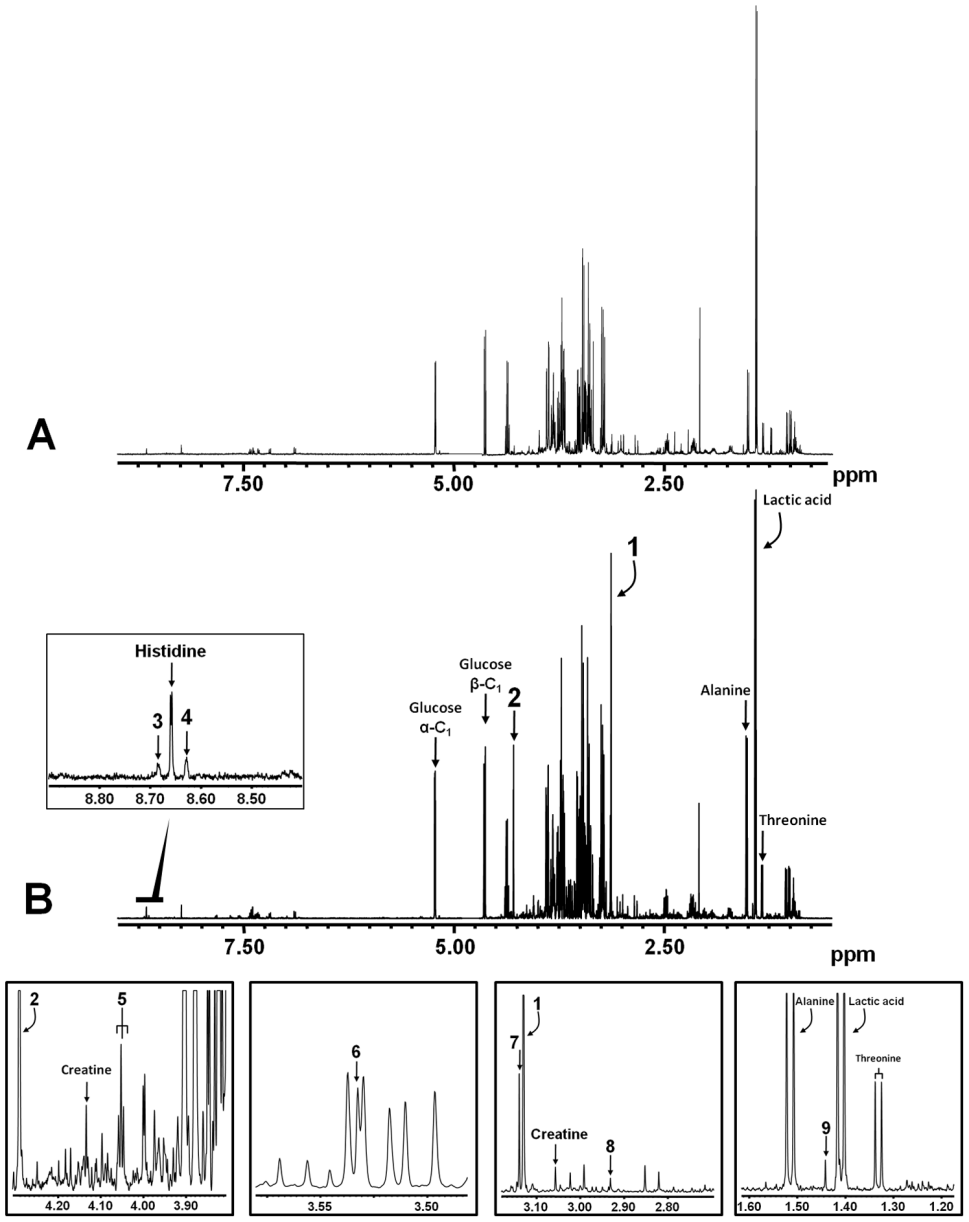
Accumulation of uremic solutes. 500 MHz ^1^H-NMR spectrum of plasma (ultrafiltrate) from (**A**) a healthy control and (**B**) a CKD patient. Insets show 5 regions of interest in greater detail. Metabolite abnormalities: Creatinine (1 and 2), 1-methylhistidine (3), 3-methylhistidine (4), *myo*-Inositol (5), trimethylamine *N*-oxide (6), dimethyl sulphone (7), *N,N*-dimethylglycine (8) and 2-hydroxyisobutyric acid (9).

^1^H-NMR spectroscopy revealed that a plethora of uremic compounds are elevated in CKD patients as compared with healthy controls (Figure 3). Resonance assignments were based on previously recorded spectra and a total of 14 solutes could be assigned. As expected, creatinine was elevated in all patients. Moreover, the well-known toxins 3-methylhistidine, hippuric acid, p-cresyl sulphate, *N,N*-dimethylglycine, betaine and *myo*-inositol were detected in all patients. In all ten patient samples we also detected the hitherto unknown toxins DMSO_2_ and 2-HIBA. 1-methylhistidine was detected in nine patients, trigonelline and trimethylamine *N*-oxide were found in six patients and pseudouridine was found in five patients; whereas, dimethylamine was only detected in one individual. Resonance assignments and concentrations of uremic toxins measured by ^1^H-NMR are summarized in Table 2 and chemical structures can be found in Figure S1.

### Comparison with Normal Concentrations

**Figure 4:**
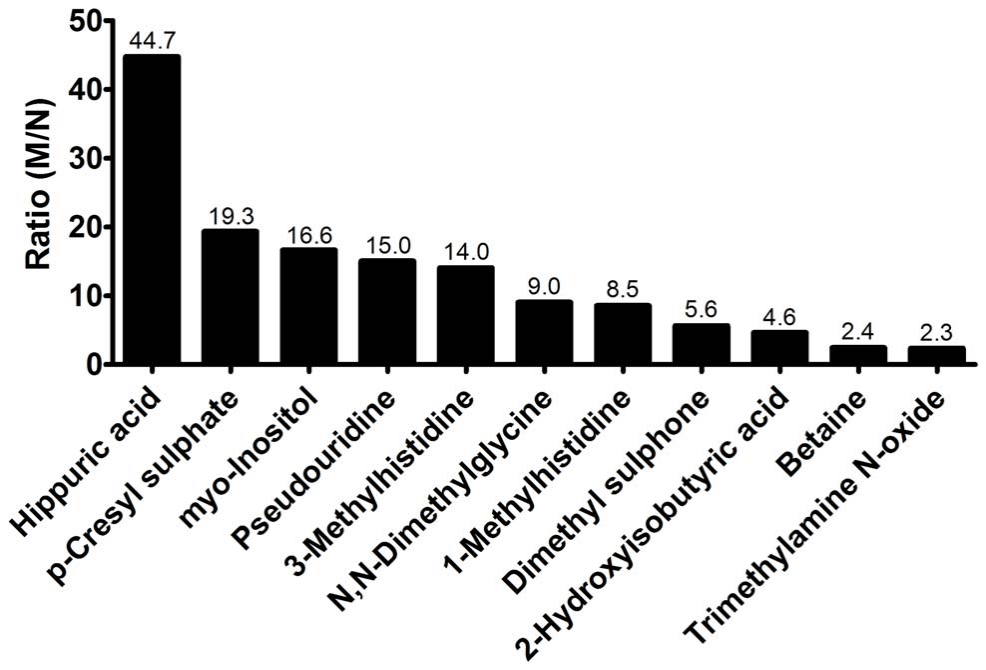
Relative retention of uremic solutes in stage 3–4 CKD patients. The M/N index is the ratio of the mean uremic concentration (M) found in the present study to the normal concentration (N) measured in healthy controls reported in literature.

To evaluate the relative solute retention in stage 3–4 CKD patients, the ratio of the mean of all uremic concentrations (M) determined were calculated to the normal concentration (N) reported in literature (M/N), as described previously [5]. The solute solely retained in one patient (*i.e.* dimethylamine), trigonelline (due to lack of reference value) and creatinine were excluded from this analysis. The M/N ratio ranged from 2.3 for trimethylamine *N*-oxide to 44.7 for hippuric acid (Figure 4). In the case of five metabolites, the uremic concentration was more than 10 times higher than normal. A moderate degree of retention was observed for four solutes for which the M/N ratio ranged between 4 and 10.

### Correlation between eGFR and Retention of DMSO_2_ and 2-HIBA

**Figure 5:**
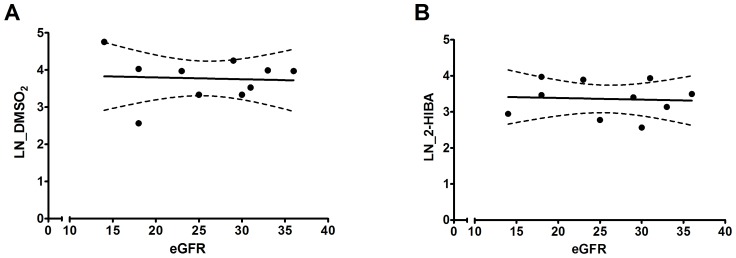
Correlation between plasma solute levels and eGFR. Dots represent the natural logarithm (LN) of individual concentrations of DMSO2 and 2-HIBA and the lines the best fit linear regression line with the 95% confidence interval.

Next, we investigated whether the plasma concentration of DMSO_2_ and 2-HIBA in non-dialysis CKD patients correlated with a decline in kidney function as represented by the eGFR. As shown in both Figure 5 and Figure S2, there is no association between the parameters studied as concluded from a Spearman correlation analysis (r<0.2 for both solutes).

### Influence of Uremic Solutes on ciPTEC Phenotype

**Figure 6:**
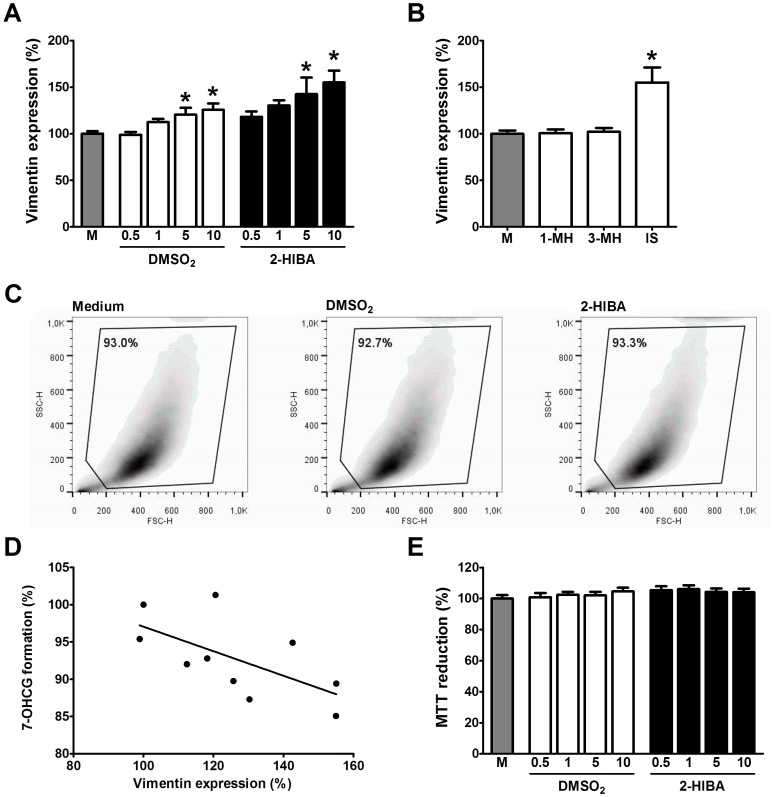
Impact of DMSO_2_ and 2-HIBA on ciPTEC. Cells were exposed for 48 h to ciPTEC medium (gray bar), DMSO_2_ or 2-HIBA (concentration range: ½ C_max_–10x C_max_). (**A**) Following treatment, cells were harvested and stained with mouse-α-human Vimentin-PE. Quantification of staining was done with a BD FACSCalibur flow cytometer using channel FL-2, and analyzed with FlowJo software, gating on live cells. Statistical analysis was performed via a One-way ANOVA followed by the Dunnett's Multiple Comparison Test for each toxin. Results are presented as mean ± SEM of three independent experiments performed in duplicate or triplicate. * indicates p<0.05 compared with control. (**B**) Vimentin expression following exposure to 1 mM 1-methylhistidine (1-MH), 3-methylhistidine (3-MH; both negative control) or indoxyl sulphate (IS; positive control) for 48 h. Results are presented as mean ± SEM of three independent experiments performed in duplicate or triplicate. * indicates p<0.05 compared with control. (**C**) Cells were exposed for 48 h to ciPTEC medium, DMSO_2_ or 2-HIBA (both 10x C_max_). Representative density plots with percentage of gated (*i.e.* living) cells of three independent experiments, performed in duplicate or triplicate (**D**) Following treatment, ciPTEC were incubated for 3 h with 10 µM 7-OHC. Afterwards, an aliquot of culture medium was collected and injected into the HPLC-system. Standards of 7-OHCG were also analyzed in order to quantify the amount of glucuronide found in the samples. Acquired HPLC data were processed with PC1000 software (Spectrasystem). Pearson correlation analysis revealed a significant association between the expression of vimentin and glucuronidation (r = −0.63; p<0.05). (**E**) The MTT assay was used to study the impact of DMSO_2_ and 2-HIBA on mitochondrial metabolism. Cells were exposed for 48 h to both solutes as described above. Afterwards, cells were incubated for 4 h with MTT-solution at 37°C. Subsequently, produced formazan crystals were dissolved in DMSO and extinction was measured at 570 nm. Results are presented as mean ± SEM of three independent experiments performed minimally in triplicate.

The impact of the novel uremic retention solutes on proximal tubular epithelium was investigated using a unique human proximal tubule cell line, demonstrated to be a valid model to study nephrotoxicity and renal cell metabolism [17]–[19]. Exposure of ciPTEC to the C_max_ of DMSO_2_ or 2-HIBA, determined in this study (Table 2), resulted in an increase in vimentin expression by 12% and 30%, respectively (Figure. 6A). Moreover, at the highest concentration tested (10x C_max,_) these toxins increased vimentin expression by 26% and 55%, respectively. In comparison, both 1-methylhistidine and 3-methylhistidine did not affect vimentin expression; whereas the positive control (indoxyl sulphate) increased vimentin expression by 55% (Figure 6B). Flow cytometry revealed that exposure to the highest concentration of both solutes did not affect cell morphology nor the percentage of living cells as compared to untreated cells (Figure 6C). Furthermore, when ciPTEC were exposed to the C_max_, UGT activity was reduced by 8% and 13%, respectively. And we observed a clear correlation between vimentin expression and UGT activity with a calculated Pearson r of −0.63 (p<0.05; Figure 6D). In addition, DMSO_2_ and 2-HIBA did not impede mitochondrial succinate dehydrogenase activity as demonstrated with the MTT assay (Figure 6E). Taken together, our findings suggest that both solutes induce a loss of epithelial characteristics and reduce renal glucuronide formation, indicating changes in cell metabolism without affecting cell viability.

## Discussion

Accumulation of uremic toxins due to inadequate renal clearance is a hallmark of CKD. Uremic retention solutes are associated with disease progression and the myriad of pathologies observed in dialysis patients. In this study, ^1^H-NMR spectroscopy was successfully used to indentify multiple uremic toxins in the plasma of stage 3–4 CKD patients.

Our results revealed that ultrafiltration and acetonitrile extraction are complementary deproteinization techniques and both are required as sample preparation methods for the proper detection of uremic retention solutes using ^1^H-NMR spectroscopy. In the study of Tiziani *et al*., it was demonstrated that ultrafiltration was the best deproteinization strategy to remove proteins from serum samples resulting in a high metabolite retainment and reproducibility [20]. Furthermore, they reported that following acetonitrile extraction most of the metabolites were maintained, although with a reduced signal intensity compared to ultrafiltration [20]. In contrast, Daykin *et al*., demonstrated that deproteinization using acetonitrile at physiological pH resulted in an increased detection of low-molecular-weight metabolites and a improved signal-to-noise ratio [11]. These studies corroborate our notion that multiple deproteinization strategies are needed when investigating the metabolome.

Here, we report for the first time that DMSO_2_ and 2-HIBA are retained in CKD patients. Moreover, both solutes were demonstrated to negatively influence renal cell physiology using ciPTEC. Already in 1966, Williams *et al*. reported the presence of DMSO_2_ in urine [21]. Yet, it took several decades to establish that DMSO_2_ is a common metabolite present in blood and cerebrospinal fluid [22], [23]. DMSO_2_ can originate from dietary sources such as, milk and port wine [23]–[25]. Moreover, it can be formed during bacterial metabolism of methanethiol in the gut or endogenous human methanethiol metabolism [23], [26]. Recently, it has been described that DMSO_2_ attenuated both constitutive as well as IL-1β-induced IL-6 and IL-8 production in human chondrocyte cell line, possibly by inhibiting the ERK1/2 signaling pathway [27]. Older studies indicated that DMSO_2_ inhibited oxidant production in activated neutrophils and diminished the proliferation of vascular smooth muscle cells and endothelial cells [28], [29]. Regarding 2-HIBA, two recent studies reported that this compound is constitutively present in human urine and serum [30], [31]. Moreover, 2-HIBA is the major urinary metabolite in humans following exposure to the gasoline additives methyl-*tert*.butyl ether and ethyl-*tert*.butyl ether [32], [33]. Elevated plasma concentrations of 2-HIBA were observed in patients with type 2 diabetes mellitus, possibly due to disturbances in fatty acid metabolism [34]. Moreover, a recent genome-wide association study revealed a significant negative association with urinary 2-HIBA levels and SNP rs830124, an intronic SNP of the *WDR66* gene on chromosome 12, which is closely associated with mean platelet volume [35], [36]. At present, little is known regarding the biological activity of DMSO_2_ and 2-HIBA. Our results provide the first proof that these metabolites induce a loss of defined renal epithelial features and are possibly involved in the progression of CKD. However, more research is necessary to definitely label these solutes as uremic toxins.

It is known that uremic toxin levels rise in concordance with CKD severity [2]. Therefore, it is suggested that eGFR, as a marker of kidney function, should reflect retention state and the levels of uremic solutes in CKD patients. In this study, we did not observe any correlation between eGFR and plasma concentration of DMSO_2_ or 2-HIBA, which suggests that eGFR is a poor marker for the accumulation of these solutes. These findings are in agreement with previous studies demonstrating that eGFR is an unsuitable tool to predict levels of uremic toxins from different classes (*e.g.* middle weight and protein-bound) in CKD patients [37], [38]. Furthermore, these results suggest that the renal clearance of both metabolites is mainly dependent on active tubular transport, possibly due to binding of the compounds to plasma proteins. Yet, more research is needed to fully elucidate the chemical properties, including protein-binding, of DMSO_2_ and 2-HIBA.

Next to DMSO_2_ and 2-HIBA, the present study showed that 1-methylhistidine, 3-methylhistidine, hippuric acid, p-cresyl sulphate, *N,N*-dimethylglycine, pseudouridine, betaine, *myo*-inositol and trimethylamine *N*-oxide were elevated in stage 3–4 CKD patients. Previously, Choi *et al*., used ^1^H-NMR spectroscopy to study the metabolic status of dialysis patients [39]. They reported that a multitude of uremic toxins were retained including *myo*-inositol and 3-methylhistidine, similar to our findings in non-dialysis CKD patients. In addition, Rhee *et al*. studied the metabolomic profile of 44 hemodialysis patients using three different LC-MS methods [4]. In total, 40 metabolites were elevated at baseline in patients, compared to healthy controls. In concordance with our results, they demonstrated retention of hippuric acid, trimethylamine *N*-oxide and dimethylglycine. Both studies did not report the retention of 1-methylhistidine, pseudouridine and betaine. However, accumulation of 1-methylhistidine in patients treated with hemodialysis has been demonstrated by HPLC previously [40]. And Niwa *et al*., reported that pseudouridine levels were increased in both CRF and dialysis patients compared with controls [41]. Taken together, there is a clear overlap in the solutes retained in both the early stages of CKD and patients with end-stage renal disease.

Previously, using HPLC and LC-MS/MS, our group demonstrated that indole-3-acetic acid, indoxyl sulfate and kynurenic acid accumulated in CRF patients with mean concentrations of 4.4 µM, 67 µM and 0.6 µM, respectively [2]. These compounds were not identified during the present study due to the limited sensitivity of proton NMR spectroscopy. Other widely deployed techniques, such as HPLC and LC-MS/MS, have a higher sensitivity with detection limits in the nano- or even picomolar range although the sensitivity highly varies depending on the compound of interest. The present study was an initial proof-of-concept study to determine whether ^1^H-NMR spectroscopy could be used as a tool to expand our knowledge about uremic retention solutes and if this technique can be utilized in the search for CKD biomarkers. As such, a small number of CKD patients was included, which limited our power to identify uremic retention solutes for which levels greatly differ among individual patients. Still, the current study is the first to provide an overview of the metabolome of non-dialysis CKD patients by means of NMR spectroscopy. One has to take into account that, due to the reasons stated above, not all of the potential toxic solutes retained in stage 3–4 CKD patients are identified. Further studies with additional subjects and analytical techniques should provide a more complete overview of metabolites retained in CKD patients.

In conclusion, a hallmark of renal failure is the retention of a plethora of metabolites, belonging to multiple physico-chemical classes, with potential deleterious effects on total body homeostasis. In this study, we have demonstrated that both ultrafiltration and acetonitrile extraction are required as deproteinization methods to elucidate the metabolomic profile of stage 3–4 CKD patients by means of ^1^H-NMR spectroscopy. Moreover, using this technique, we have successfully identified a total of 14 metabolites, including 2 novel uremic solutes, that possibly contribute to the co-morbidity and mortality in CKD patients. These results might aid in revealing new biomarkers for CKD and possibly contribute to a better understanding of the progressive character of renal disease.

## Supporting Information

Figure S1 Chemical structures of uremic solutes detected in stage 3–4 CKD patients. Structures obtained from the Human Metabolome Database (www.hmdb.ca) [42]. (TIF)

Figure S2 Correlation between plasma solute levels (µM) and eGFR. Dots represent the individual concentrations of DMSO_2_ and 2-HIBA and the lines the best fit linear regression line with the 95% confidence interval. Nonparametric Spearman correlation analysis revealed no significant correlation between the two studied parameters for both DMSO_2_ (r = −0.17; p = 0.6) and 2-HIBA (r = −0.03; p = 0.9). (TIF)
