# Supplementary figures and images for: Optimized Metabolomic Approach to Identify Uremic Solutes in Plasma of Stage 3–4 Chronic Kidney Disease Patients
Source: PLoS One. 2013 Aug 2;8(8):e71199. doi: 10.1371/journal.pone.0071199 (PMC3732267; doi:10.1371/journal.pone.0071199)

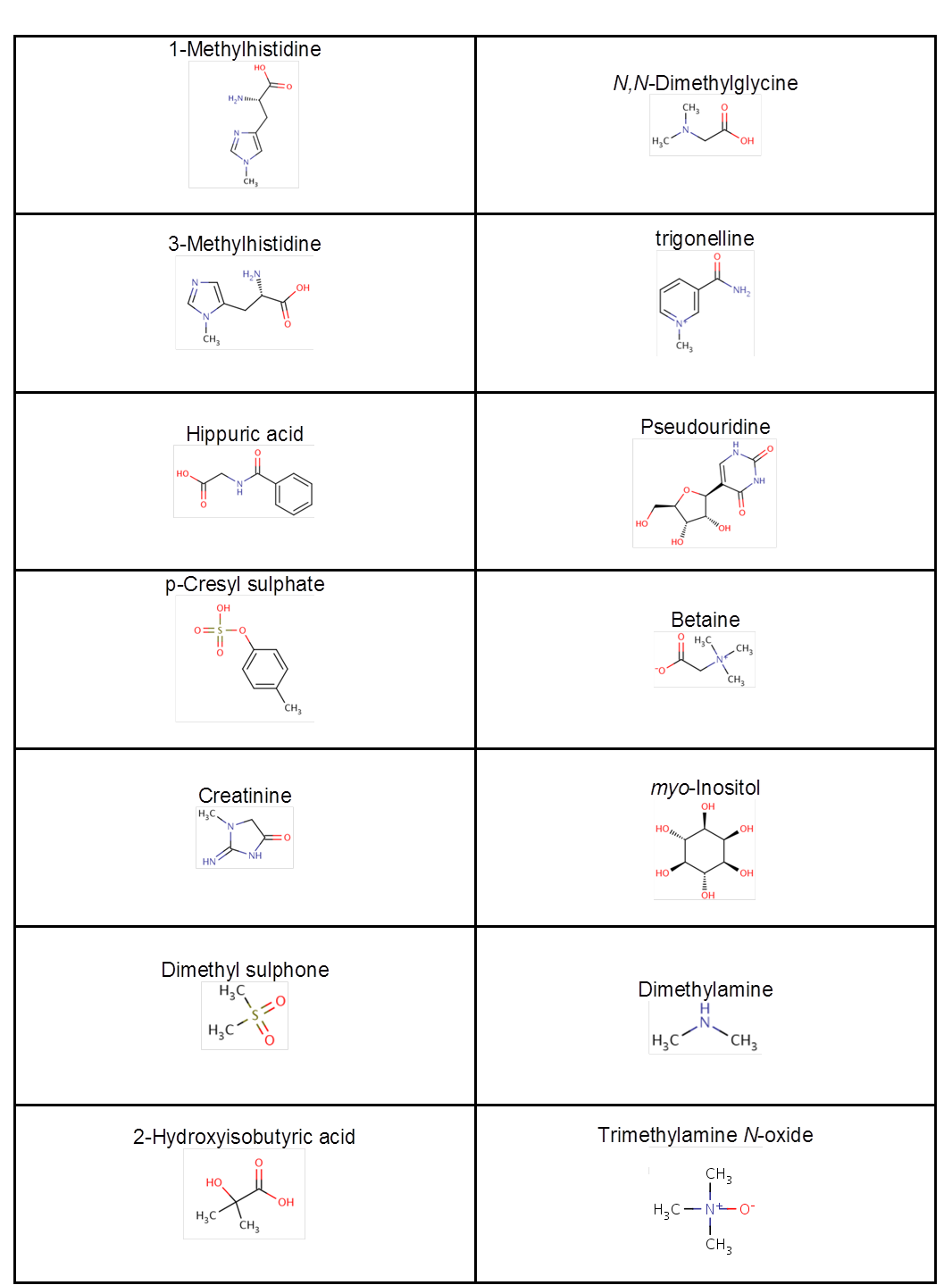

Supplement: Figure S1 — (TIF) [file pone.0071199.s001.tif]

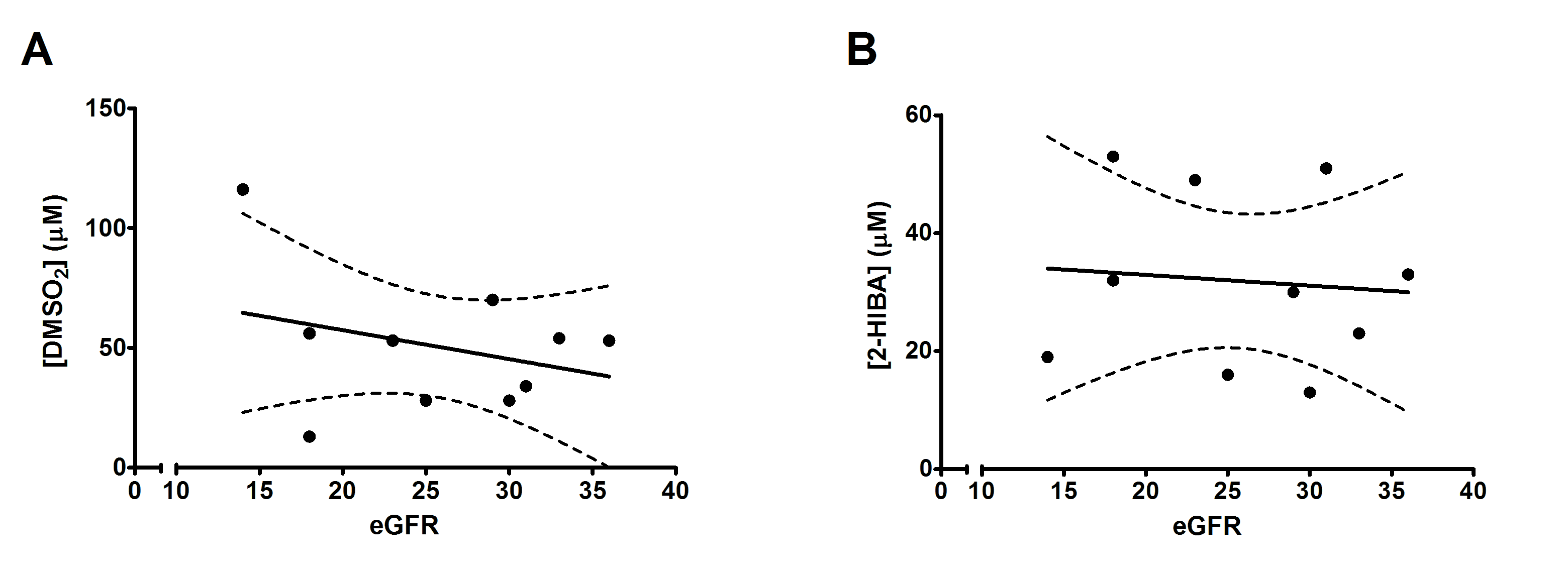

Supplement: Figure S2 — (TIF) [file pone.0071199.s002.tif]
